# Justification of Body Mass Index cutoffs for hip and knee joint arthroplasty among California orthopedic surgeons

**DOI:** 10.1186/s13018-025-05551-3

**Published:** 2025-01-31

**Authors:** Sophie V. Orr, Gavin C. Pereira, Blaine A. Christiansen

**Affiliations:** Department of Orthopaedic Surgery, Lawrence J. Ellison Musculoskeletal Research Center, University of California Davis Health, 2700 Stockton Blvd, Suite 2301, Sacramento, CA 95817 USA

**Keywords:** BMI, Arthroplasty, Osteoarthritis, Weight stigma

## Abstract

**Background:**

Many orthopedic surgeons choose not to perform joint arthroplasty on patients with a Body Mass Index (BMI) of 35 or above, citing poorer outcomes and increased procedure risk. Identifying and addressing factors surgeons use to determine procedure BMI cutoffs are necessary to increase access to orthopaedic care for this growing patient population. This will help reduce healthcare disparities while also identifying clinical facilities, equipment, training, and procedures that require improvements to accommodate larger individuals.

**Methods:**

Orthopaedic surgeons were surveyed to identify surgeon-specific BMI cutoffs for hip and knee arthroplasty. The survey was circulated within the California Orthopaedic Association (COA) report during March 2023. Questions aimed to identify BMI cutoffs and justifications such as infection risk, co-morbidities, inadequate equipment, and the American Academy of Orthopaedic Surgeons (AAOS) guidelines. Data on decision making about BMI cutoffs and exceptions were also collected.

**Results:**

75% of respondents use BMI cutoffs for hip and knee arthroplasty. 91% of respondents indicated they are either wholly or partially responsible for setting procedure BMI cutoffs. Mean hip and knee arthroplasty BMI cutoffs were 40.5 and 41, respectively. Four categories for BMI cutoff justifications were identified: (1) risk of complications; (2) surgery logistics; (3) concerns about facilities or resources; and (4) surgeon perception.

**Conclusions:**

BMI-based justifications for denial of care define key addressable areas of improvement that can increase access to care for life-changing orthopaedic surgeries such as THA and TKA. Insight from the queried surgeons will help drive future research areas to address this need.

## Introduction

The average weight of Americans has been increasing over the past several decades [1–4], and so is the demand for orthopaedic surgeries [5–7]. However, patients with larger body sizes may not have the opportunity to pursue some elective orthopaedic procedures. Many orthopaedic surgeons have BMI limits, or cutoffs, for elective surgeries, citing poorer outcomes and increased procedure risk [8–11]. These risks can include increased infection rate, cardiac conditions, adverse reaction to anesthesia, and other life-threatening complications.

Total Hip Arthroplasty (THA) and Total Knee Arthroplasty (TKA) procedures are mainly conducted on patients with severe osteoarthritis (OA), many of whom have higher body weight as high body mass has been identified as a risk factor for OA [12, 13]. Access to THA and TKA surgeries can be limited at the discretion of surgeons and medical facilities based on multiple risk factors. One of these risk factors is the Body Mass Index (BMI) of the patient. BMI was originally known as the Quetelet Index after the Belgian polymath who developed it in the 1830s to define the average man [14]. These statistics were based on Western European men of the time and did not consider other genders, ethnicities, or health factors [14, 15]. Ancel Keys [16, 17] rebranded the Quetelet Index to the Body Mass Index (BMI) in 1972 to support his nutritional assertions. It was later adopted by health insurance companies [18] and medical professionals as a quantitative metric to gauge the overall health of a patient [17, 18]. BMI is still being used today by many orthopaedic surgeons with the effect of restricting access to life-changing surgeries such as THA and TKA.

BMI cutoffs for THA and TKA are often as low as 30 or 35 [19]. Previous research and surveys have identified that risk of infections and complications are a major reason for these cutoffs [19], but do not go into specifics that can help drive research directions. The American Academy of Orthopaedic Surgeons (AAOS), the major professional organization for orthopedic surgeons in the US, recommends that THA and TKA surgeries should have a BMI cutoff of 40 [20]. The reason for differences between the AAOS guidelines and practicing California surgeon BMI cutoffs is unknown. To investigate the specifics of this discrepancy and the prevalence of BMI cutoffs in the state, we designed a survey for distribution to members of the California Orthopaedic Association (COA). The survey audience was orthopaedic surgeons who regularly perform THA and/or TKAs. This study does not aim to identify if a patient should lose weight, but rather what barriers exist that prevent a patient at a higher weight in undergoing THA or TKA from a qualified orthopaedic surgeon. The questions were focused on identifying BMI cutoffs, pinpointing justifications for these cutoffs, and organizing these justifications into actionable categories to help reduce access to care disparities for individuals with a BMI over 35. With insight from these practicing orthopaedic surgeons, this study aimed to identify future areas of research that can increase access to care and clinical outcomes for millions. Specifically, we wanted to (1) discover the prevalence of BMI cutoffs; (2) identify BMI cutoff decision-making authorities; (3) identify currently used BMI Cutoffs, where applicable; and (4) document the BMI Cutoff Justifications used by California orthopaedic surgeons.

## Materials and methods

### Survey design and distribution

This survey was designed to identify key factors impacting access to hip and knee arthroplasty based on patient BMI in California. It was optimized to meet this goal with guidance from the UC Davis Health School of Medicine Office of Research Evaluation unit. The survey was pretested by an experienced orthopaedic surgeon (GCP) and questions were modified for clarity. The University of California, Davis Institutional Review Board determined this research was exempt from IRB approval as no personal identifying information or patient information was collected. The questions were distributed as a cross-sectional survey in Qualitrics (Qualtrics XM, Seattle, WA) within the California Orthopaedic Association (COA) report, the weekly COA newsletter, during March 2023. Members who had signed up to receive email alerts received this email. The report is also available online to the public on the COA Report website. This study included only California surgeons because it was designed to generate preliminary data from surgeons in our region that would later be used to inform future nationwide studies of BMI cutoff usage and justifications.

This survey consisted of 13 questions, the first of which asked about the specific setting of each respondent’s surgical practice. Subsequent questions were aimed at gauging the prevalence and degree of BMI cutoffs currently employed by Californian orthopaedic surgeons for THA and TKA. Years of practice, whether the surgeons use BMI cutoffs, and the respective BMI limits for surgeries were requested. To identify reasons surgeons used BMI cutoffs, the survey asked who is wholly or partially responsible for BMI decisions for both THA and TKA, as well as what factors or justifications were used to determine these limits. Multiple selections were allowed via a “select all that apply” instructions, and additional factors could be added through an “other: please specify” option. Justifications were categorized in the analysis as a framework for future investigation on this topic. All responses were required except for one which asked for any additional comments respondents would like to offer. Full survey questions are available in Appendix A.

#### Collected responses

Out of 39 respondents, 33 completed the questionnaire (84.6% completion rate). Therefore, this analysis includes data from 32 respondents who reported that they performed hip and/or knee arthroplasties. One respondent stated that they did not perform those surgeries, so their responses were not included in the analysis.

#### Data analysis

Data analysis was conducted on Microsoft Excel (Redmond, WA, USA) and Prism 10 (La Jolla, CA, USA).

## Results

### Prevalence of BMI cutoffs

**Fig. 1:**
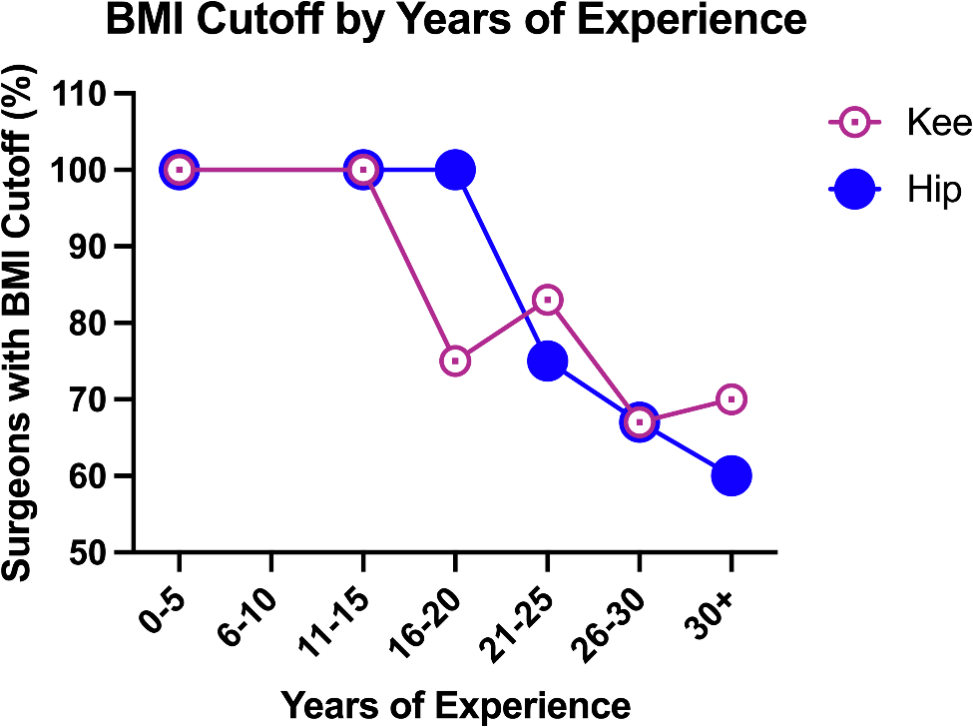
Percent of Californian orthopaedic surgeons with BMI cutoffs for knee and hip arthroplasties as a function of years of practice. (Abbreviations: BMI: Body Mass Index)

Overall, 75% of respondents that perform THA and/or TKA self-reported using BMI cutoffs. Surgeons with more years of experience were less likely to use BMI cutoffs for these surgeries (Fig. 1). All respondents with 15 or fewer years of experience used BMI cutoffs for both THA and TKA. For surgeons with 16–20 years of experience, all used BMI cutoffs for THA, but only 75% used cutoffs for TKA. A majority of surgeons with over 20 years of experience used BMI cutoffs for both THA and TKA, but the prevalence of BMI cutoff use tended to decrease with more years of experience.

### BMI cutoff decision making

**Fig. 2:**
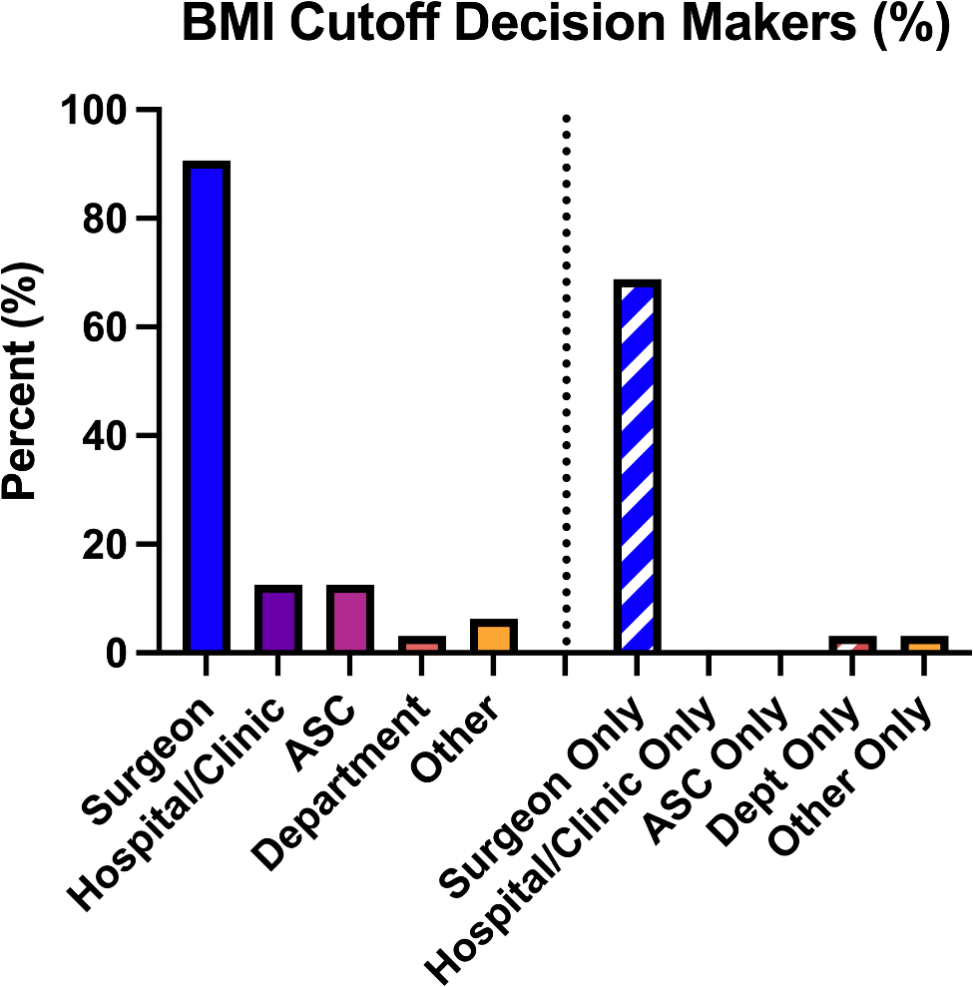
Who determines BMI cutoffs for hip and knee joint arthroplasties in California. (Abbreviations: BMI: Body Mass Index, ASC: Ambulatory surgery center)

Surgeons were asked, in general for both surgeries, about their BMI cutoff decision making. 91% of surgeons said they had partial or total decision-making power about whether to use a BMI cutoff for THA and TKA surgeries and what cutoff number to use (Fig. 2). 69% reported the cutoff was at their sole decision. Only 6% of respondents listed their department or another entity as the sole decision makers.

### BMI cutoffs numbers

**Fig. 3:**
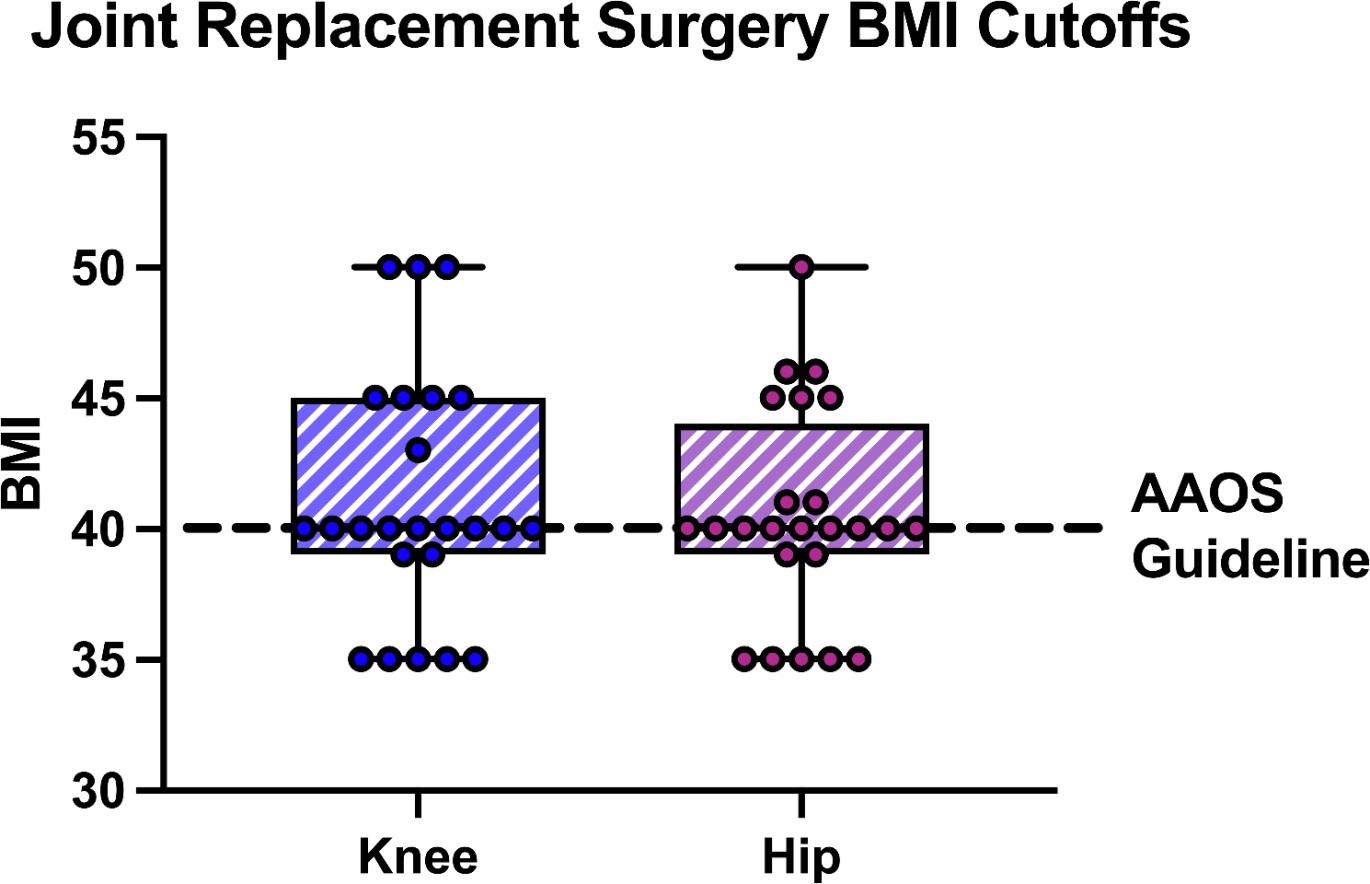
Self-reported BMI Cutoffs of Californian Orthopaedic Surgeons as they compare to the AAOS BMI guidelines. (Abbreviations: BMI: Body Mass Index, AAOS: American Academy of Orthopaedic Surgeons)

Mean THA and TKA BMI cutoffs were 40.5 and 41, respectively (Fig. 3). Many surgeons used BMI cutoff numbers that were higher or lower than suggested by the AAOS.

### BMI cutoff justifications

**Fig. 4:**
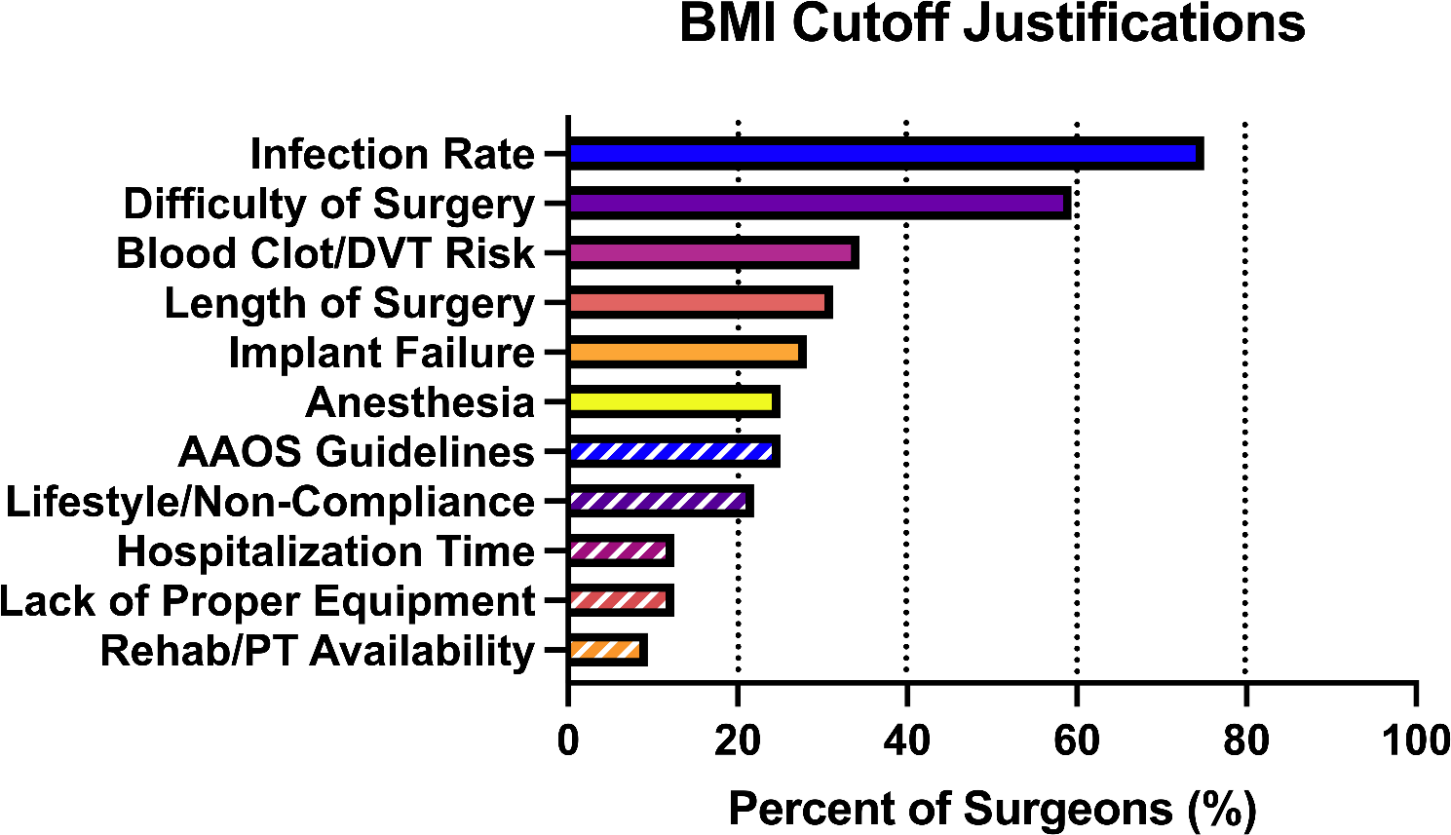
BMI cutoff justifications for Californian orthopedic surgeons performing hip and knee arthroplasties. (Abbreviations: BMI: Body Mass Index, DVT: Deep vein thrombosis, AAOS: American Academy of Orthopaedic Surgeons)

Surgeons were asked to identify justifications in their BMI cutoff decisions for both THA and TKA (Fig. 4). The most common justifications identified by respondents were infection rate (75%) and difficulty of surgery (59%). Other justifications based on complications with surgery included blood clot/DVT risk (38%) and concerns about anesthesia (25%). Other concerns with performing the surgery included the increased length of surgery (30%). Justifications based on surgeon perception included the risk of implant failure (27%) and concerns about patient lifestyle or non-compliance (21%). Relatively few respondents were concerned about facilities or resources including hospitalization time (15%), lack of proper equipment (15%), and rehab/PT availability (9%).

**Fig. 5:**
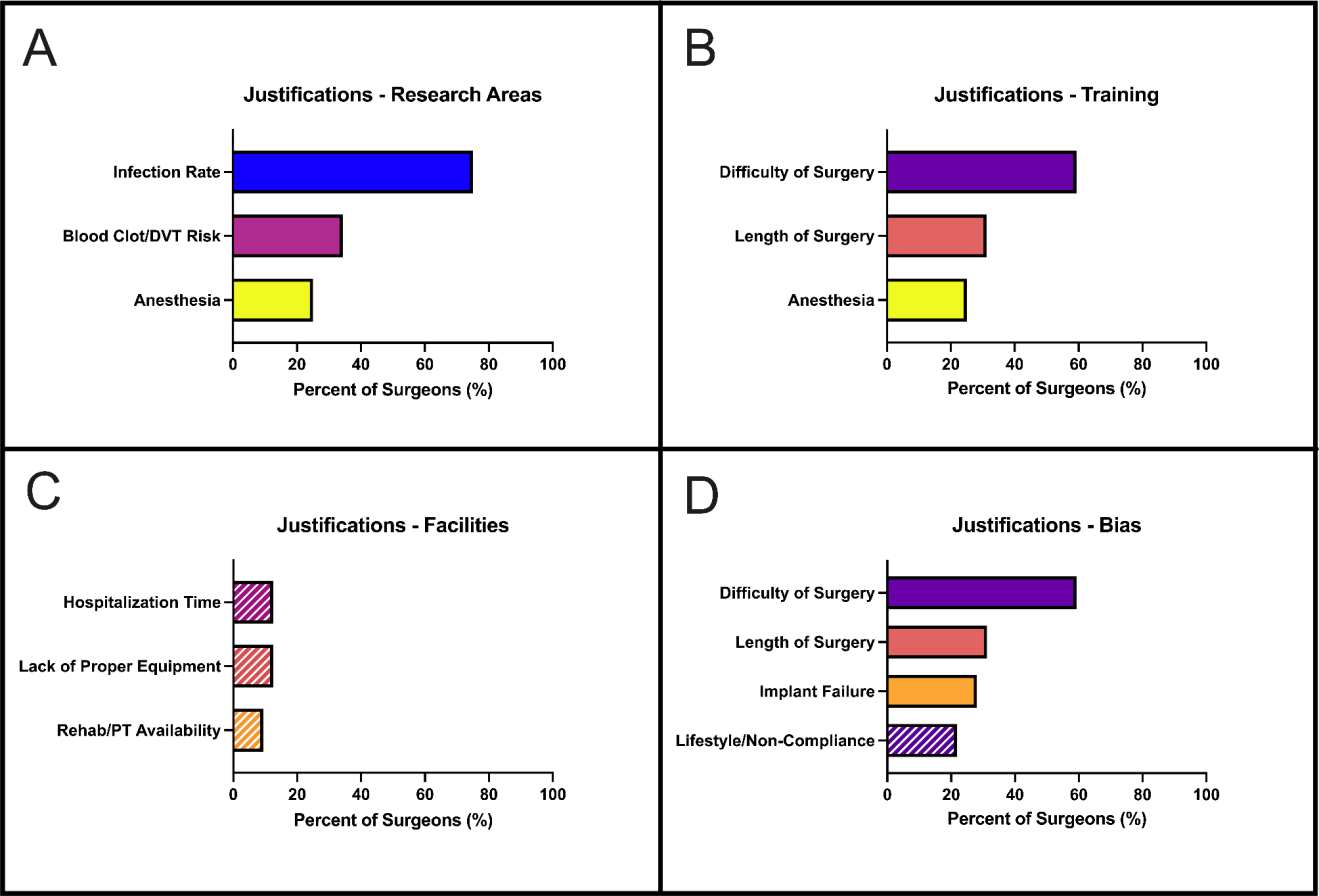
Categorized BMI Cutoff justifications for Californian orthopedic surgeons performing hip and knee arthroplasties. Four categories for BMI cutoff justifications were identified: **(A)** increased risk of complications, **(B)** logistics for performing surgeries, **(C)** concerns about facilities or resources, and E) surgeon perception. (Abbreviations: BMI: Body Mass Index, DVT: Deep vein thrombosis, AAOS: American Academy of Orthopaedic Surgeons)

For further analysis, these justifications were categorized (Fig. 5). This included possible areas to be address by future research (Fig. 5A), issues that could be addressed with additional training (Fig. 5B), issues that could be addressed with updated facilities and resources (Fig. 5C), and issues that may be due to surgeon perception or bias (Fig. 5D).

## Discussion

In this study we surveyed California orthopaedic surgeons who regularly perform THA and/or TKA about BMI cutoffs for these procedures. This preliminary survey was only circulated among members of the California Orthopaedics Association, so surgeons practicing THA and TKA in California that were not part of this organization may not be properly represented. Additionally, these responses may not represent the policies and decision making of surgeons outside of California. The survey received a small number of responses compared to the number of orthopaedic surgeons in California. However, the COA website lists 295 “total joint” surgeons in their member directory, leading us to believe our 32 responses is a large enough proportion of relevant surgeons (> 10%) to glean important information regarding BMI cutoffs for these surgeries. Through their responses, we were able to assess the prevalence and specific cutoff numbers for these procedures. We also identified four categories for BMI cutoff justifications including (1) increased risk of complications, (2) logistics for performing surgeries, (3) concerns about facilities or resources, and (4) surgeon perception.

Our resulting average values (BMI cutoffs of 40.5 and 41 for THA and TKA, respectively) are in line with another survey distributed nationally to the American Association of Hip and Knee Surgeons Research Committee members, though the western/Pacific northwest portion of their respondents had a markedly lower BMI cutoff than present in our data [19]. The Western/Pacific northwestern portion makes up just 13% of their total study population, so their aggregated findings may not represent Californian surgeons.

### Research Gaps

Justifications for denying a THA or TKA due to a high BMI pertaining to surgery-related complications represent possible areas for future research (Fig. 5A). Medical complications can arise during surgery, perioperatively, and post-operatively with varying levels of severity. Database studies frequently compare the rate of complications as a function of BMI category (underweight, normal weight, overweight, and the obesity classes). Complications that correlate with increased BMI can include cardio-respiratory issues, infection, and the need for revisions [21]. Surgery-related issues can lead to longer hospital stays and increased resource utilization and cost of care [22, 23], but not in all cases [23, 24]. Additionally, the literature currently has no consensus on the prevalence of these issues after THA and TKA in different BMI categories. Certain clotting conditions, such as pulmonary embolism, exhibit a higher likelihood in individuals with elevated BMI, whereas others, like deep vein thrombosis, remain unaffected by variations in patient weight [25]. Readmission and infections, including surgical site infections and perioperative joint infections, are one of the most identified risks of high BMI individuals [23, 26–29], though the correlation of BMI and infection risk is not clear for all surgeons or operations [30]. There is also no consensus as to the impact of BMI on THA and TKA on overall functional outcomes [23, 31–33]. The pro-inflammatory state in an high BMI patient puts them at a higher risk of Perioperative cardiac and respiratory complications [34]. Due to the metabolic syndrome seen in bariatric patients, there is an increased risk of cardiac morbidity and mortality, in general, but this has not been defined in the joint arthroplasty population due to the extremely low mortality rates. Similarly, respiratory challenges in high BMI patients during and after surgery include hypoxia, need for higher positive end-expiratory pressure (PEEP), higher incidence of obstructive sleep apnea (OSA) and higher risk of intensive care unit (ICU) admissions [35]. There is some evidence to suggest that operative times are also increased in patients with higher BMI [21, 36].

Orthopaedic research may be able to close several knowledge gaps that prevent safe surgeries on high BMI individuals including infection rate, blood clot/DVT risk, and adverse effects of anesthetics. Understanding the mechanisms that increase these issues in people with higher body weight, whether biological or procedure based, will help advance translational research that benefits patients of all sizes. As the average BMI of Americans increases, new research should be conducted to apply treatments of past studies to larger body sizes. Future medical studies and clinical trials should include a range of BMIs to reflect the impact of these treatments on a representative sample of patients that represent all patient populations.

### Training

Perceived difficulty of surgery, increased length of surgery, and adverse effects of anesthetics on high BMI patients may be addressable by increased and specific training on caring for patients with larger body sizes (Fig. 5B) [34]. With practice, surgery difficulty and length can be reduced, and anesthesia application can be improved. Additionally, the negative correlation between years of experience and percentage of surgeons implementing BMI cutoffs for joint arthroplasty could represent a variety of training-related issues. These concerns could indicate that medical schools and residency programs are not adequately preparing trainees for operating on high BMI individuals, requiring years of post-graduation and post-residency experience to adequately serve these patients. This is consistent with research about bias in medical schools and those training in healthcare fields [37]. Additionally, it is a possibility that the more experienced surgeons can handle the complexities of the surgery better than their younger counterparts or that they are just not aware of the AAOS guidelines (or chose to ignore them because of lived experience).

### Facilities

Hospitalization time, lack of proper equipment, and rehabilitation/physical training availability were less influential on a surgeon’s chosen BMI cutoff (Fig. 5C). However, these factors are still a systemic concern for hospitals around the US [38–40]. Developing a system to share equipment between orthopaedic surgery and bariatric surgery centers could help address any concerns related to access to appropriate facilities and equipment.

### Surgeon Perception and Bias

In some cases, surgeons being unable or unwilling to operate on higher BMI individuals may be influenced by implicit or explicit anti-fat bias. Concerns about lifestyle and assumed non-compliance after surgery indicate an unspoken moral standard has been applied to high BMI patients (Fig. 5D). These prejudices heavily impact patient wellbeing beyond denial of care [41–44]. Patients that experience weight stigma have worse outcomes and morbidities compared to high BMI patients that have not experienced this stigma [44]. Increased body size may add difficulty or length to surgery, but these concerns would likely be mitigated with proper training and resources. Length of surgery as a justification to avoid high BMI joint arthroplasty patients may also reflect anti-fat bias. A metadata analysis of over 5 million patients found that mean surgery times were increased for high BMI patients by just 6 min for all surgeries (from 83 to 89 min) and 7 min for orthopaedic surgeries (from 76 to 83 min) [21]. THA and TKA surgeries carry a much shorter operation time than other elective orthopaedic procedures, and length of surgery can be impacted by many factors beyond weight, including inter-surgeon variability, available support staff, and even day of the week [45–47]. Inadequate training, education, and facilities, as well as provider bias, all impact the ability of high BMI individuals to receive life-changing healthcare. This is a growing issue in many medical fields. As the population weight increases, so will the medical costs associated with delayed or avoided medical care. A scarcity of orthopaedic surgeons that are able and willing to perform surgeries on larger individuals will increase these issues unless policies and medical training are modified to be more accommodating of high BMI patients.

There is currently no consensus about the impact of BMI on implant failure based on implant survival and surgery outcomes [10, 31, 48–51]. Studies have found that there is no difference in knee prosthesis failures or outcomes regarding BMI or total body weight [10, 50, 51]. Others suggests that high BMI demonstrate increased infection rates in THA and TKA, and that reduced outcomes may be due to higher BMI at younger ages [52, 53]. Research has demonstrated increased dislocations of hip arthroplasty related to BMI [48, 49], but that mechanical failure and aseptic loosening were not correlated with BMI [49]. Several implant manufacturers include high BMI as a contradiction for the use of their products, though some researchers have shown that the forces on an implant reduce with height more so than weight, so BMI based cutoffs may be unjustified with respect to implant failure [31]. Despite these conflicting studies, the majority acknowledge that joint arthroplasty surgeries still provide a vast improvement to the quality of life of high BMI patients. THA and TKA surgeries carry extremely high success and satisfaction rates compared to other elective surgeries, so while many factors go into a surgeon’s decision about whether to operate on a patient, concerns about implant failure should not be primary factor affecting this decision.

### Professional Organization guidelines

We found that less than 1 out of 4 California orthopaedic surgeons considered the national American Association of Orthopaedic Surgeons (AAOS) guidelines as a key factor for their BMI cutoff selection. Current AAOS guidelines cite reduced clinical outcomes for individuals with high BMI based on “strong evidence”. However, a review of the few provided publications cited on the AAOS OrthoGuildelines website at the time of publication [54, 55] demonstrated conflicting evidence. BMI/Obesity received their highest risk factor recommendation of “Strong Recommendation” for TKA based on the articles cited on these pages. However, several of these publications stated that outcomes are not correlated with BMI [10, 50, 54–56]. For example, Judge et al. determined that there were no reduced outcomes in higher BMI individuals, and that BMI should not be an access to care barrier given the benefits of TKA [50, 51]. The main risk factors listed for high BMI individuals were wound complications, such as surgical site infections, and reduced functional outcomes. However, outcomes such as Knee Society scored were still higher for high BMI patients post-TKA than their pre-clinical range and function scores [11], demonstrating that this surgery still provided an improvement in function for these individuals. The AAOS and similar professional organizations should consider revisiting their BMI cutoff guidelines and justifications to better mirror current understandings in the field, such as those cited by this study, which some Californian surgeons are currently using to inform their own BMI cutoffs.

## Conclusions

Access to THA and TKA for high BMI individuals in California is primarily determined by the orthopaedic surgeons that choose whether or not to perform their surgery. These orthopaedic surgeons, as well as the medical facilities where they practice, have an opportunity to provide life-changing relief to osteoarthritis patients suffering from pain and mobility issues. Improvements in training, access to more accommodating facilities, updated institutional policies, bias training, and orthopaedic research aimed at addressing surgical complications in high BMI individuals may increase equitable access to healthcare for high BMI patients.

## Appendix A: Orthopaedic Surgeon patient BMI survey

Questions distributed as a Qualtrics study to the COA:

1. Do you perform knee and/or hip replacement surgeries?

- Yes.
- No.

2. In what setting do you practice?

Select all that apply.

- Hospital - General.
- Hospital – Private.
- Hospital – Military.
- Hospital - University/Academic.
- Hospital - Ambulatory Surgery Centers (ASC).
- Private Practice - Single surgeon practice.
- Private Practice - Group practice.
- Private Practice - Hospital-based.
- Other (fill in).

3. In what locality do you practice?

Select all that apply.

- Urban.
- Suburban.
- Rural.

4. How many years have you been performing knee and/or hip replacement surgeries?

- 0–5.
- 6–10.
- 11–15.
- 16–20.
- 21–25.
- 26–30.
- 30+.

5. How many knee replacement surgeries do you conduct per year, on average?

- 0–10.
- 11–25.
- 26–50.
- 51–100.
- 101–150.
- 151–300.
- Over 300.

6. How many hip replacement surgeries do you conduct per year, on average?

- 0–10.
- 11–25.
- 26–50.
- 51–100.
- 101–150.
- 151–300.
- Over 300.

7. In general, what BMI cutoffs do you have for the following surgeries?

Your BMI cutoff is the highest BMI of a patient you would perform the surgery on based on their BMI. For example, if you would operate on a patient with a BMI of 34 but not 35, your BMI cutoff is 34.

Select “No Cutoff” if you do not have a BMI Cutoff.

- Slider from 20 to 60 for knee surgeries and a “No Cutoff” check box.
- Slider from 20 to 60 for hip surgeries and a “No Cutoff” check box.

8. What factors influenced the BMI cutoff decision?

Select all that apply.

- Infection rate.
- Blood clot/DVT rate.
- Difficulty of surgery for the surgeon.
- Anesthesia.
- Lifestyle or non-compliance issues.
- Implant failure.
- Post-op hospitalization time.
- Rehabilitation/physical therapy availability.
- Access to proper equipment for large patients.
- AAOS guidelines.
- Other (fill in).

9. Are there exceptions to your normal BMI cutoff points?

If yes, please elaborate.

- Yes (fill in).
- no.

10. Who determines the BMI cutoff for knee and hip replacement surgeries you perform?

Please select all that apply.

- The Surgeon (you).
- The department.
- The hospital/clinic.
- Ambulatory Surgery Center (ASC).
- Other.

11. On average, how many knee replacement patients do you refer to other treatments/surgeons because of their BMI each year?

- 0–10.
- 11–25.
- 26–50.
- 51–100.
- 101–150.
- 151–300.
- Over 300.
- I do not see patients with a BMI over my cutoff.

12. On average, how many hip replacement patients do you refer to other treatments/surgeons because of their BMI each year?

- 0–10.
- 11–25.
- 26–50.
- 51–100.
- 101–150.
- 151–300.
- Over 300.
- I do not see patients with a BMI over my cutoff.

13. Do you have any comments relating to the content of this survey?

If yes, please elaborate.

- Yes (fill in).
- no.

## Data Availability

The datasets used and/or analyzed during the current study are available from the corresponding author on reasonable request.
